# Mapping the CLEC12A expression on myeloid progenitors in normal bone marrow; implications for understanding CLEC12A‐related cancer stem cell biology

**DOI:** 10.1111/jcmm.13519

**Published:** 2018-02-07

**Authors:** Marie Bill, Peter B. van Kooten Niekerk, Petter S. Woll, Laura Laine Herborg, Anne Stidsholt Roug, Peter Hokland, Line Nederby

**Affiliations:** ^1^ Department of Hematology Aarhus University Hospital Aarhus Denmark; ^2^ Department of Medicine Center for Hematology and Regenerative Medicine Karolinska Institutet Stockholm Sweden; ^3^ Department of Hematology Aalborg University Hospital Aalborg Denmark; ^4^ Department of Clinical Immunology and Biochemistry Lillebaelt Hospital Vejle Denmark

**Keywords:** CLEC12A protein, haematopoiesis, hMICL, immunophenotyping, myeloid progenitor cells, neoplastic stem cells

## Abstract

The C‐type lectin domain family 12, member A (CLEC12A) receptor has emerged as a leukaemia‐associated and cancer stem cell marker in myeloid malignancies. However, a detailed delineation of its expression in normal haematopoiesis is lacking. Here, we have characterized the expression pattern of CLEC12A on the earliest stem‐ and myeloid progenitor subsets in normal bone marrow. We demonstrate distinct CLEC12A expression in the classically defined myeloid progenitors, where on average 39.1% (95% CI [32.5;45.7]) of the common myeloid progenitors (CMPs) expressed CLEC12A, while for granulocyte‐macrophage progenitors and megakaryocyte‐erythroid progenitors (MEPs), the average percentages were 81.0% (95% CI [76.0;85.9]) and 11.9% (95% CI [9.3;14.6]), respectively. In line with the reduced CLEC12A expression on MEPs, functional assessment of purified CLEC12A^+/−^ CMPs and MEPs in the colony‐forming unit assay demonstrated CLEC12A^+^ subsets to favour non‐erythroid colony growth. In conclusion, we provide evidence that the earliest CLEC12A^+^ cell in the haematopoietic tree is the classically defined CMP. Furthermore, we show that CLEC12A‐expressing CMPs and MEPs are functionally different than their negative counterparts. Importantly, these data can help determine which cells will be spared during CLEC12A‐targeted therapy, and we propose CLEC12A to be included in future studies of myeloid cancer stem cell biology.

## 1 INTRODUCTION

The transmembrane receptor C‐type lectin domain family 12, member A (CLEC12A) is emerging as a marker of blasts and leukaemic stem cells (LSC) in acute myeloid leukaemia (AML) and by inference as a useful tool in both the diagnostic and follow‐up settings.^1^, ^2^, ^3^, ^4^, ^5^ In addition, we have recently shown the relevance of this marker in the context of myelodysplastic syndrome (MDS).^6^ In the setting of malignant haematology, one of the most appealing features of CLEC12A is its lack of expression on CD34^+^CD38^−^ cells in normal bone marrow (BM), regenerating BM and G‐CSF‐stimulated peripheral blood stem cells,^2^ making it a potential treatment target. Recent ventures in this regard include the development of anti‐CD3/anti‐CLEC12A bispecific antibodies.^7^, ^8^, ^9^ The upcoming roles of CLEC12A as a cancer stem cell marker and potential treatment target emphasize the need of knowing the details of its expression pattern on early stem and progenitor cells in healthy individuals.

Although the classical roadmap of haematopoiesis is constantly being revised,^10^, ^11^, ^12^ it is nevertheless providing a powerful tool for understanding the production of mature circulating blood cells and continuously serves as a solid model for studying clonal evolution in the myeloid spectrum of haematological malignancies.^13^, ^14^, ^15^, ^16^ In this study, we opted to employ the immunophenotypic definition of the haematopoietic stem cell (HSC) as being Lin‐CD34^+^CD38^−^CD90^+^CD45RA^−^,^17^, ^18^, ^19^ the multipotent progenitor (MPP) as being Lin‐CD34^+^CD38^−^CD90^−^CD45RA^−^ 20 and the human multilymphoid progenitor (MLP, also termed lymphoid‐primed multipotent progenitor) as Lin‐CD34^+^CD38^−^CD90^−^/lowCD45RA^+^.^21^ With respect to the Lin‐CD34^+^CD38^+^ myeloid progenitors, we distinguished the common myeloid progenitor (CMP), the granulocyte‐macrophage progenitor (GMP) and the megakaryocyte‐erythroid progenitor (MEP) subsets based on their CD123 and CD45RA expression as first described by Manz and colleagues.^22^

Using these definitions, we have delineated the expression of CLEC12A on normal BM stem‐ and myeloid progenitor cells and evaluated the influence of this marker on the growth of purified CMPs, GMPs and MEPs in the colony‐forming cell assay. Importantly, our results provide evidence that the earliest CLEC12A^+^ cell in normal haematopoiesis is the classically defined CMP.

## 2 METHODS

### 2.1 Bone marrow samples

Bone marrow samples (n = 13) from healthy volunteers were obtained in the setting of BM harvest for allogeneic BM transplantation or donated as part of the anonymous biobank for normal donors in Hemodiagnostic Laboratory, Department of Hematology, Aarhus University Hospital. The biobank is approved by the local Ethics Committee of the Central Denmark Region, and sample collection was performed after informed consent was given. Mononuclear cells (MNCs) were obtained by Lymphoprep (Axis‐Shield plc., Dundee, Scotland) separation according to the manufacturer's instructions, cryopreserved in 10% dimethylsulphoxide and stored in liquid nitrogen.

### 2.2 Flow cytometry

Cryopreserved MNCs were thawed in 37°C water bath and resuspended in RoboSep Buffer (StemCell Technologies, Vancouver, BC, Canada) with 15% heat‐inactivated foetal calf serum (Biochrom, GmbH, Berlin, Germany). Subsequently, cells were stained with the monoclonal antibodies (MoAb) listed in Table 1. Lineage depletion was accomplished by introducing a dump channel with lineage‐MoAbs. Data acquisition was performed on a Navios flow cytometer (Donor 1‐9) (Beckman‐Coulter, Inc., Brea, CA, USA) equipped with three lasers; 405 nm, 488 nm and 638 nm, respectively. Compensation was set using with UltraComp eBeads (eBioscience, San Diego, CA, USA) together with the relevant fluorochrome‐conjugated antibodies. Data were analysed with FlowJo Data Analysis Software, version X (FlowJo, Ashland, OR, USA). For each sample and for each fluorochrome, positive and negative gates were determined by means of fluorescence minus one (FMO) controls.

**Table 1:** Applied fluorochrome‐conjugated monoclonal antibodies

MoAb	Fluorochrome	Clone	#Cat	Company
CD45RA	FITC	HI100	304106	BioLegend
CLEC12A	PE	HB3		Own lab
CD38	ECD	LS198.4.3	A99022	BC
CD2	PE‐Cy5	RPA‐2.10	300209	BioLegend
CD3	PE‐Cy5	HIT3a	300309	BioLegend
CD4	PE‐Cy5	RPA‐T4	300509	BioLegend
CD7	PE‐Cy5	CD7‐6B7	343110	BioLegend
CD8a	PE‐Cy5	RPA‐T8	301009	BioLegend
CD10	PE‐Cy5	HI10a	312206	BioLegend
CD11b	PE‐Cy5	ICRF44	301307	BioLegend
CD14	PE‐Cy5	RMO52	A07765	BC
CD19	PE‐Cy5	HIB19	302209	BioLegend
CD20	PE‐Cy5	2H7	302307	BioLegend
CD56	PE‐Cy5	B159	555517	BD
CD235a	PE‐Cy5	HIR2	306605	BioLegend
CD123	PE‐Cy7	6H6	306010	BioLegend
CD34	APC	BIRMA‐K3	C7238	DAKO
CD90	BV421	5E10	328122	BioLegend

FITC, fluorescein‐isothiocyanate; PE, phycoerythrin; ECD: PE‐Texas Red PE‐Cy5: phycoerythrin‐cyanine5; PE‐Cy7, phycoerythrin‐cyanine7; APC, allophycocyanin; BV421, brilliant violet 421. BD, BD Biosciences; BC, Beckman‐Coulter.

**Figure 1:**
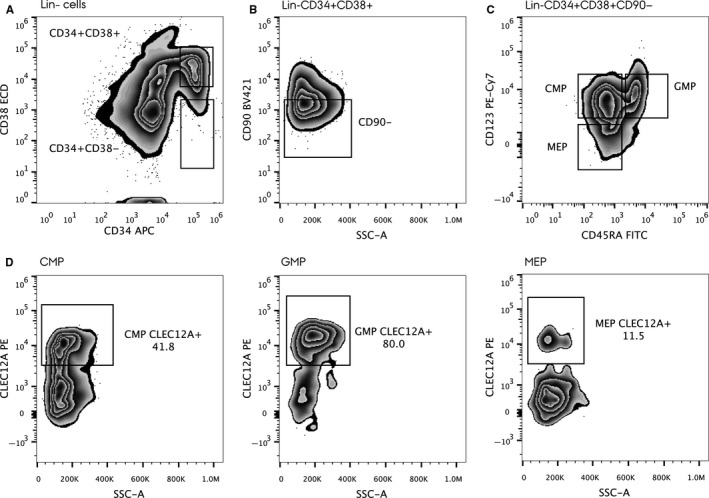
Gating strategy in the myeloid progenitor compartment. Initially, cells were gated in a forward scatter—side scatter plot, and the Lin‐ events were selected (not shown). In the Lin‐ cell population, the CD34^+^ CD38^+^ cells were identified (A) and depicted in a CD90 vs. side scatter plot (B). To further enrich for progenitors, the CD90‐ cells were selected and depicted in a CD123 vs. CD45RA plot, whereby the CMP, the GMP and the MEP could be identified (C). These myeloid progenitor subsets were then further gated for their CLEC12A expression (D)

The gating strategy used for analysing the myeloid progenitor cells is depicted in Figure 1. In short, the lineage negative singlets were defined (not shown) and displayed in a CD34 vs. CD38 plot. Herein, the CD34^+^CD38^+^ cell subset was defined (Figure 1A) and the progenitors were further enriched by CD90 negativity (Figure 1B). The CMPs were defined as being Lin‐CD34^+^CD38^+^CD90^−^CD123^+^CD45RA^−^, GMPs as Lin‐CD34^+^CD38^+^CD90^−^CD123^+^CD45RA^+^ and MEP as Lin‐CD34^+^CD38^+^CD90^−^CD123^−^CD45RA^−^ (Figure 1C). Next, these three myeloid progenitor subsets were further gated by their CLEC12A expression (Figure 1D). The gating strategy used for analysing CLEC12A expression on HSCs, MPPs and MLPs in the Lin‐CD34^+^CD38^−^ subset is depicted in Figure S1. The number of analysed events and the corresponding percentages of CLEC12A^+^ cells are given in Table S1.

### 2.3 Fluorescence‐activated cell sorting

Cryopreserved BM MNCs from 6 donors (D1, D2, D10, D11, D12 and D13) were thawed and stained with the MoAbs listed in Table 1. Fluorescence‐activated cell sorting was performed on a BD FACSAria™ III (BD Biosciences) equipped with four lasers; 405 nm, 488 nm, 561 nm and 633 nm, respectively. Using the same gating strategy as described above, the CMPs, GMPs and MEPs were sorted into CLEC12A^+/−^ subsets, respectively. Due to the rarity of the sorted cell populations, post‐sort purity was determined in 21 of 36 cases and reported in Table S2. Flow cytometry data from the sorting experiments (Donor 10‐13) were included in the overall analyses of CLEC12A expression on myeloid progenitors.

### 2.4 Colony‐forming cell assay

Up to 500 sorted cells from subsets representing CLEC12A^+/−^ CMPs, GMPs and MEPs were seeded in 35‐mm discs in 1 ml of MethoCult H4435 (StemCell Technologies) supplemented with penicillin‐streptomycin (Gibco, Thermo Fischer Scientific Inc.) and allowed to incubate for 14 days at 37°C, in 95% humidity and 5% CO_2_. Whenever possible, cells were seeded in duplicates. Colony‐forming cells were counted and scored by morphology as burst‐forming unit‐erythroid (BFU‐E), colony‐forming unit‐erythroid (CFU‐E), colony‐forming unit‐granulocyte‐erythroid‐macrophage‐megakaryocyte (CFU‐GEMM), colony‐forming unit‐granulocyte‐macrophage (CFU‐GM), colony‐forming unit‐macrophage (CFU‐M), and colony‐forming unit‐granulocyte (CFU‐G). For the seeded GMPs, a clear distinction between CFU‐GMs, CFU‐Ms and CFU‐Gs was possible and colonies were counted as such. For CMPs and MEPs, the morphology of myeloid colonies was less clear; hence, CFU‐GMs, CFU‐Ms and CFU‐Gs were counted together as myeloid colonies. The cellular composition of each colony type was verified by May‐Grünwald Giemsa staining (Sigma‐Aldrich, St. Louis, MO, USA) of representative colonies cytospun onto slides (n = 1 (donor 1); data not shown).

### 2.5 Statistical analyses

All calculations were conducted in GraphPad Prism version 6 (GraphPad Software, La Jolla, CA, USA). The D'Agostino & Pearson omnibus test was used to test whether the percentage of CLEC12A^+^ cells followed a Gaussian distribution. For comparison of the CLEC12A^+^ cell subsets between CMP, GMP and MEP, Student's unpaired t test was used. The Mann‐Whitney test was applied to test differences in colony formation in CLEC12A^+/−^ progenitor subsets. A two‐sided *P*‐value < .05 was considered significant.

## 3 RESULTS

### 3.1 CLEC12A is differentially expressed on myeloid progenitor subsets

**Figure 2:**
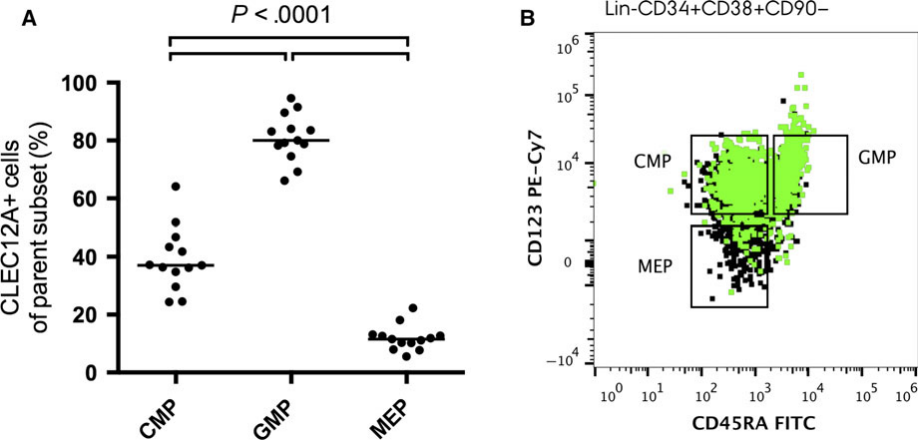
Distribution of CLEC12A^+^ cells in myeloid progenitors. Percentage of CLEC12A^+^ cells in CMP, GMP and MEP in BM from 13 healthy donors. Horizontal bars denote the mean. The *P*‐value applies to all three comparisons (A). Overlay of the CLEC12A^+^ cell population on the Lin‐CD34^+^ CD38^−^ CD90^−^ cells displayed in a CD45RA vs. CD123 plot showing the distribution of the CLEC12A^+^ progenitors (B)

To study at what level in the early human haematopoiesis CLEC12A is expressed, we employed a seven‐colour tube (Table 1) and applied a stringent negative gating strategy based on FMO controls (Figure 1, S1). Consistent with previous observations,^2^ we did not detect CLEC12A expression on either the HSCs or the MPPs in the Lin‐CD34^+^CD38^−^ subset (Table S1). For the MLP subset, the number of events was very low, and thus, it cannot formally be ruled out that a fraction of these cells could express CLEC12A (Table S1). The mean percentage of CMPs expressing CLEC12A was 39.1% (95% CI [32.5;45.7]), while for GMPs and MEPs, the CLEC12A^+^ fractions averaged 81.0% (95% CI [76.0;85.9]) and 11.9% (95% CI [9.3;14.6]), respectively. Hence, the mean percentage of CLEC12A^+^ CMPs was significantly different from the mean percentage of CLEC12A^+^ cells in GMPs (*P* < .0001) and MEPs (*P* < .0001). Also, the mean CLEC12A^+^ fractions were significantly different between GMPs and MEPs (*P* < .0001) (Table S1, Figure 2A). Notably, the CLEC12A^+^ cells were distributed across the whole CMP and GMP compartments and extended into the MEP compartment (Figure 2B). Taken together, while CLEC12A is a known marker of mature myeloid cells,^23^, ^24^ these data reveal that detectable expression of CLEC12A emerges at the level of the CMP, and is abundantly expressed on GMPs and to a lesser extent on the classically defined MEP.

### 3.2 Different clonogenic potential in CLEC12A positive and negative myeloid progenitors

**Figure 3:**
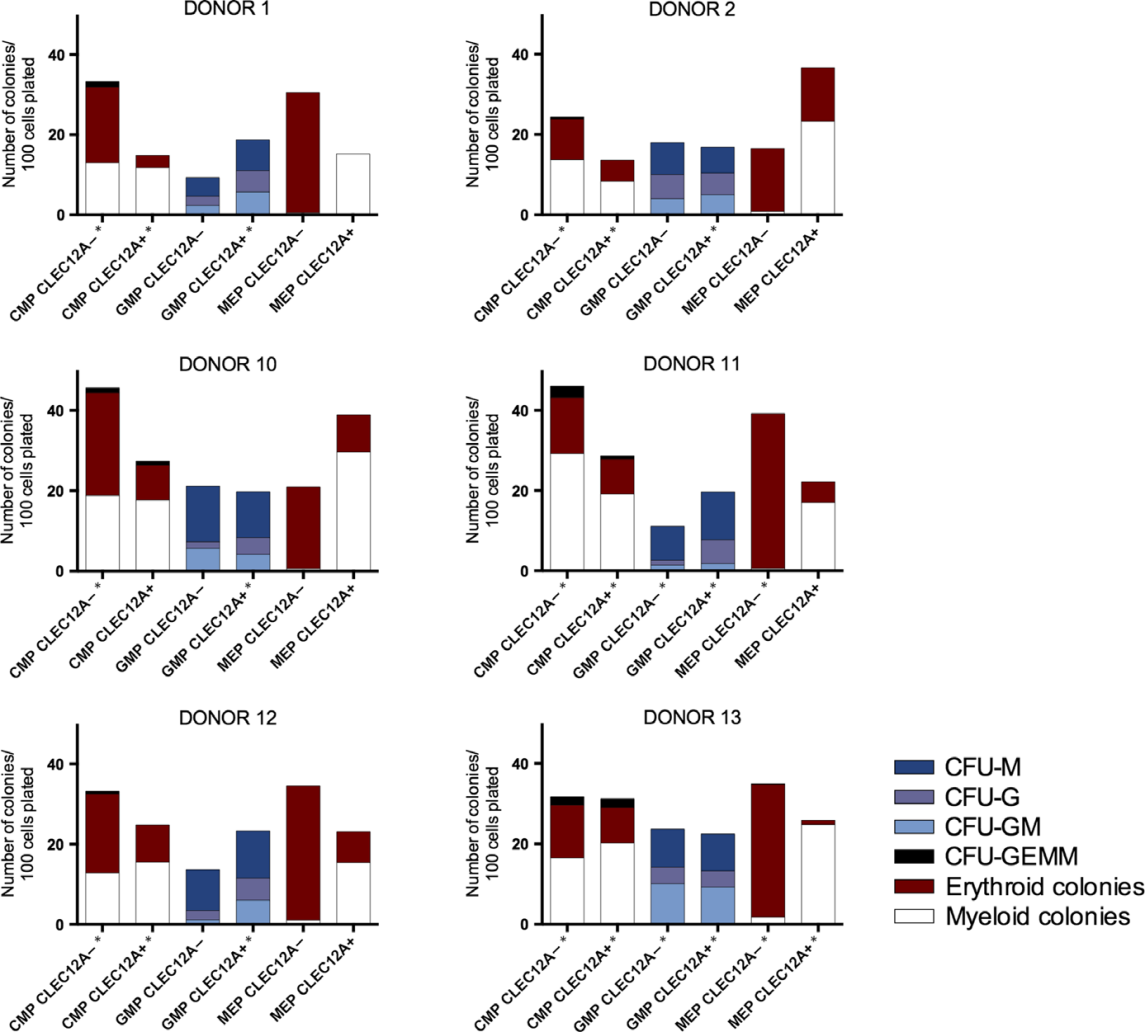
Colony‐forming cell assays on CLEC12A^+/−^ CMPs, GMPs and MEPs from 6 normal donors. Asterisk (*) denotes mean number of colonies from plates seeded in doublets.

Next, the clonogenic potential of CLEC12A positive and negative myeloid progenitors was evaluated in the colony‐forming cell assay (Table 2, Figure 3). Being the most immature of the three cell types, the CMPs gave rise to both myeloid, erythroid and CFU‐GEMM colonies in both the CLEC12A^+^ and CLEC12A^−^ subsets. The total number of colonies was significantly higher in the seeded CLEC12A^−^ CMPs (*P* = .02). Moreover, significantly fewer erythroid colonies were observed in the seeded CLEC12A^+^ CMP subset (*P* = .002), showing that CLEC12A positivity favours a non‐erythroid lineage. For both CLEC12A^+/−^ GMPs, the clonogenic growth was almost exclusively of myeloid origin and no CFU‐GEMMs were evident (Table 2), which is in accordance with the original definition of this cell subset.^22^ We found no difference neither in the overall clonogenic potential of the CLEC12A^+^ and CLEC12A^−^ GMPs (*P* = .31) nor in the distribution of the different myeloid colonies between the CLEC12A^+/−^ GMPs (CFU‐GM (*P* = .33); CFU‐M (*P* = .79); CFU‐G (*P* = .08)). With respect to MEPs, where the expected read out would be colonies of erythroid origin, we found that the CLEC12A^+^ MEPs predominantly gave rise to myeloid colonies (on average 79.8%) and the number of erythroid colonies was significantly lower among the seeded CLEC12A^+^ MEP subset compared to the CLEC12A^−^ MEP subset (*P* = .002), indicating these particular cells to represent a non‐MEP progenitor. The read out of CLEC12A^−^ MEPs was almost exclusively erythroid (on average 96.8%) as would be expected of true MEPs (Table 2). This indicates that adding CLEC12A could be useful in distinguishing true erythroid progenitors from cells with myelomonocytic potential. In conclusion, the CFU‐assays demonstrated heterogeneity in the CMP and MEP progenitor subsets when adding the CLEC12A marker to the sorting scheme with CLEC12A^+^ progenitors favouring colonies of the myelomonocytic lineage.

**Table 2:** Colony distribution of cultured myeloid progenitor subsets

CFU type	CLEC12A^−^ subset		CLEC12A^+^ subset		*P*‐value
	Median number of colonies/100 cells plated (range)	%	Median number of colonies/100 cells plated (range)	%	
CMP					
Total	32.9 (24.4‐46.0)	100	25.6 (13.7‐31.2)	100	.02
CFU‐GEMM	1.3 (0.6‐2.8)	4.0	0.45 (0‐2.2)	2.3	.18
Myeloid	15.1 (12.8‐29.2)	48.4	16.6 (8.3‐20.2)	66.5	.90
Erythroid	16.5 (10.1‐25.6)	47.6	8.8 (3.0‐9.3)	31.2	.002
GMP					
Total	15.8 (9.3‐23.7)	100	19.8 (16.9‐23.3)	100	.31
CFU‐GEMM	0.0 (0.0‐0.0)	0.0	0.0 (0.0‐0.0)	0.0	‐
Myeloid	15.8 (9.3‐23.4)	99.8	19.8 (16.9‐23.1)	99.9	.31
Erythroid	0.0 (0.0‐0.3)	0.2	0.0 (0.0‐0.2)	0.1	>.99
MEP					
Total	32.5 (16.5‐39.2)	100	24.5 (15.2‐38.9)	100	.79
CFU‐GEMM	0.0 (0.0‐0.2)	0.1	0.0 (0.0‐0.0)	0	>.99
Myeloid	0.7 (0.5‐1.8)	3.2	20.2 (15.2‐29.6)	79.8	.002
Erythroid	31.5 (15.7‐38.6)	96.8	6.5 (0.0‐13.3)	20.2	.002

CMP, common myeloid progenitor; GMP, granulocyte‐macrophage progenitor; MEP, megakaryocyte‐erythroid progenitor; CFU, colony forming unit.

## 4 DISCUSSION

This data set confirms the observation of normal CD34^+^CD38^−^ cells lacking the CLEC12A marker (Table S1, Figure S1), and by including a detailed delineation of the myeloid CD34^+^CD38^+^ progenitors, we have extended the seminal findings by van Rhenen et al.^2^ We demonstrated the consistently distinct expression of CLEC12A at the level of the classically defined CMPs, GMPs and MEPs with the marker emerging at the level of the CMP, being abundantly expressed on the GMP subset and to a lesser extent on the phenotypically defined MEPs. By investigating the CLEC12A positive and negative progenitors in the colony‐forming cell assay, we furthermore provided evidence of functional differences particularly in the CMP and MEP subsets, where CLEC12A positivity favoured colony growth of myelomonocytic lineage.

Importantly, our findings could add to the deeper understanding of CLEC12A as a blast‐ and cancer stem cell marker in the spectrum of myeloid neoplasms. Acute myeloid leukaemia has to a large extent served as a model for studying disease‐propagating cells,^25^ and while the first publications indicated the LSC to immunophenotypically resemble the normal CD34^+^CD38^−^ HSC,^26^, ^27^ recent research has indicated most cases of AML to propagate from the progenitor compartment with functional LSC activity in cells resembling the MLP^16^ and for CD34‐ AML even more mature progenitors/precursors.^15^, ^28^ In addition, in low‐risk myelodysplastic syndrome (MDS), the cancer stem cells have been shown to reside in the HSC compartment,^13^ although studies on high‐risk MDS indicate the MDS‐propagating cell to arise in the MPP or GMP compartment, much resembling AML.^29^, ^30^ In this context, the likely usefulness of CLEC12A as a marker of the leukaemic blasts and/or stem cells could be linked to the fact that the level of differentiation arrest occurs in a cell type naturally expressing this marker. By inference—regarding CLEC12A as a marker of myeloid progenitors and their downstream progeny—the presence of CLEC12A on AML blasts and stem cells could indicate a myeloid progenitor/precursor disease, while CLEC12A^−^ AML could correspond to a more immature stem cell disease originating from a cell at the level of the CLEC12A^−^ CMP or upstream. In accordance with this, CLEC12A expression on AML blasts is highly correlated with CD34 negativity,^1^ which is often associated with a more mature subtype of AML.^15^ Whether CLEC12A expression on leukaemic blasts has prognostic impact in CD34^+^ AML remains to be elucidated. Future studies are warranted to explore this in detail and also to examine whether the aberrant expression of CLEC12A on CD34^+^CD38^−^ cells shown in some cases of AML^2^, ^4^ and MDS^6^ is due to an up‐regulation of CLEC12A in these early stem/progenitor cells or a down‐regulation of CD38 in the leukaemic CLEC12A^+^ cells.

Our data impact on the notion of CLEC12A as a druggable protein in myeloid malignancies, where the first open‐label, first‐in‐human phase 1 study of a bispecific CLEC12A‐CD3 antibody has been initiated in Europe (EudraCT number 2015‐003704‐23). Thus, while such a treatment will most likely spare the earliest haematopoietic stem and progenitor cells (the Lin‐CD34^+^CD38^−^ cell subset) and thereby not affect long‐term haematopoiesis, this study provides evidence that approximately 60% of CMPs together with progenitors capable of maintaining the erythroid and—although not addressed in this work—megakaryocyte lineages will also remain unaffected by such approaches. On the other hand, given that more than 80% of GMPs would be expected to be eradicated, it cannot be excluded that a pronounced and long‐lasting depletion of functional neutrophils would follow. While CLEC12A is expressed on mature eosinophilic‐ and basophilic granulocytes,^24^, ^31^ our study did not include a functional evaluation of the eosinophil and basophil potential of the CLEC12A^+/−^ myeloid progenitors; hence, a prediction of how CLEC12A directed therapy would affect these cell types cannot be made. In addition, our results cannot formally rule out the possibility that CLEC12A could be expressed at low levels intracytoplasmic in possible myeloid primed early CD34^+^CD38^−^ haematopoietic stem‐ and progenitor cells. In fact, novel insights into human haematopoiesis question the existence of true oligo‐ and bipotent progenitors.^32^ Nevertheless, as new targeted treatment strategies directed towards surface proteins are continuously evolving, most recently supplemented with the exciting development of chimeric antigen receptor (CAR)‐engineered T cells (CAR‐T cells) directed against CLEC12A,^33^, ^34^ detailed knowledge of the surface expression of this receptor remains important in a translational setting.

Based on our findings showing a highly differentiated expression of CLEC12A on the classically defined myeloid progenitors, we propose CLEC12A could be a helpful marker to add in future detailed studies of the early human haematopoiesis. The technical advances in flow cytometry allowing the addition of more surface markers combined with novel RNA sequencing techniques and functional assays at the single cell level have opened new doors to study haematopoiesis.^35^ Indeed, Kawamura et al recently applied CLEC12A together with CD64 to study the early monocytic differentiation at the level of GMPs.^36^ In further support of adding CLEC12A in the studies of human haematopoiesis, Drissen et al studied murine myeloid differentiation pathways performed with RNA sequencing of 63 single pre‐granulocyte‐macrophage cells, and interestingly, CLEC12A turned up as a potential subpopulation classifier being one of the 55 genes showing differential expression by cluster analysis and proved to be mutually exclusive with GATA1.^37^ In this study, the GATA1^+^ (and thereby CLEC12A^−^) cells gave rise to mast cells and eosinophils, while the GATA1^−^ (CLEC12A^+^) cells gave rise to monocytes and neutrophils—cells that are also known to express CLEC12A in humans.^23^, ^24^, ^37^ Interestingly, by adding the surface markers CD71 and BAH‐1 to the established sorting schemes of myeloid progenitors, a study by Notta and colleagues found the originally defined CMPs to be quite heterogeneous and to consist of unipotent myeloid and erythroid progenitors with very sparse megakaryocyte activity.^11^ In line with this, our data indicate that adding CLEC12A as a de‐selection marker in the sorting scheme of MEPs could purify the early erythroid subset further.

In conclusion, we here show the differentiated expression of CLEC12A at the level of myeloid progenitors and provide evidence that the earliest CLEC12A^+^ cell in the haematopoietic tree is the classically defined CMP. While this knowledge is readily translational in the setting of CLEC12A‐targeted treatment, we also suggest that the marker is continuously explored in basic research studying the early myeloid differentiation.

## AUTHOR CONTRIBUTIONS

M.B., L.N., P.B.v.K.N., P.W. and P.H. designed the study. M.B., L.L.H. and L.N. performed the experiments. M.B. and L.N. analysed the data. M.B. drafted the manuscript and received input and critical reviews from L.N., P.B.v.K.N., P.H., P.W. and A.S.R.. P.H. provided the financial background for the study. All authors read and approved the final manuscript.

## CONFLICT OF INTERESTS

The authors confirm that there is no conflict of interests.
