# Supplementary figures and images for: Mapping the CLEC12A expression on myeloid progenitors in normal bone marrow; implications for understanding CLEC12A‐related cancer stem cell biology
Source: J Cell Mol Med. 2018 Feb 7;22(4):2311–8. doi: 10.1111/jcmm.13519 (PMC5867061; doi:10.1111/jcmm.13519)

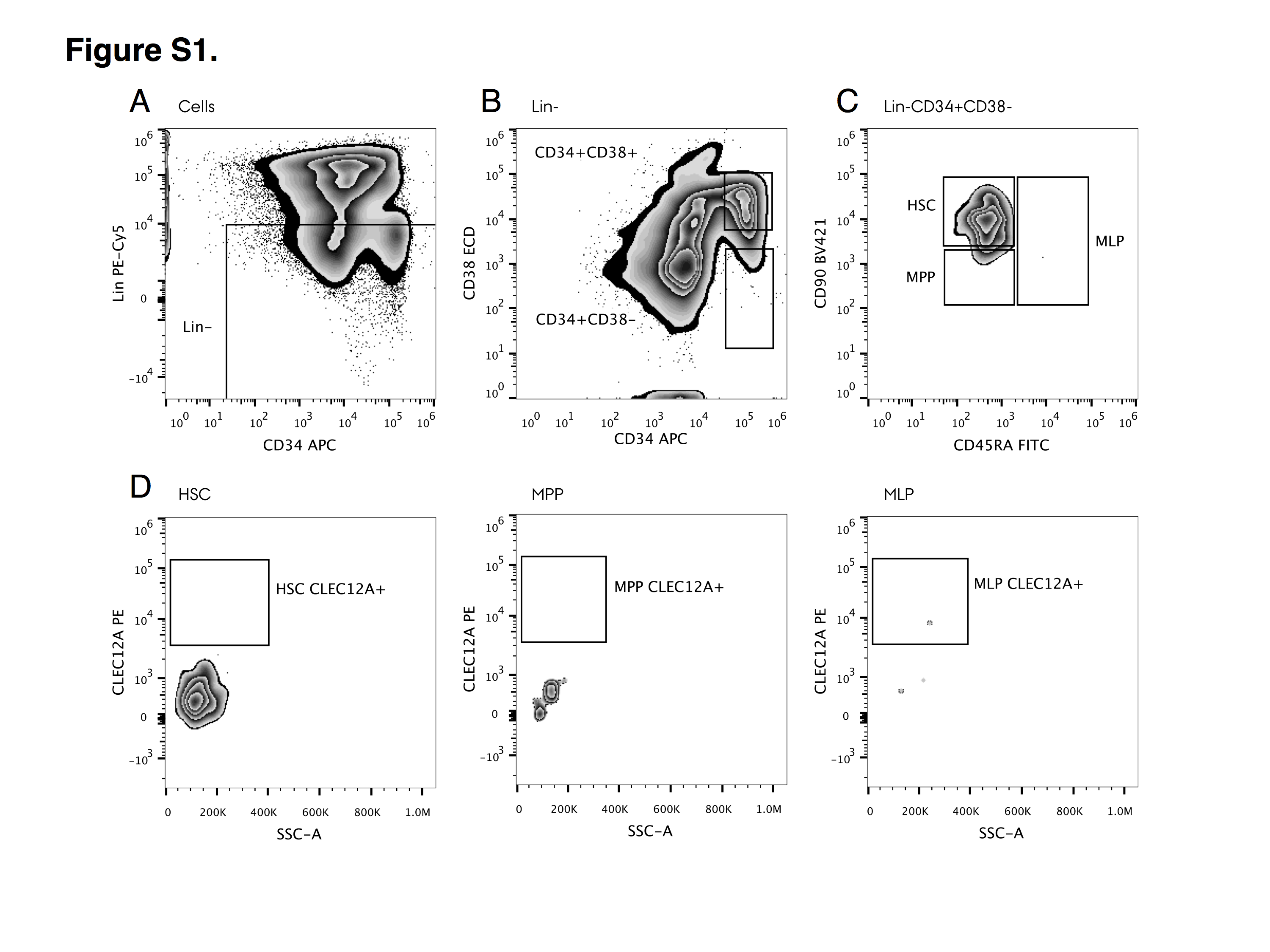

Supplement: Supplementary file 1 [file JCMM-22-2311-s001.tiff]
