# Supplementary material for: Mapping the CLEC12A expression on myeloid progenitors in normal bone marrow; implications for understanding CLEC12A‐related cancer stem cell biology
Source: J Cell Mol Med. 2018 Feb 7;22(4):2311–8. doi: 10.1111/jcmm.13519 (PMC5867061; doi:10.1111/jcmm.13519)
Supplement: Supplementary file 2 [file JCMM-22-2311-s002.docx]

| **Table S1. CLEC12A expression in early myeloid hematopoiesis** | | | | | | | | |
| --- | --- | --- | --- | --- | --- | --- | --- | --- |
|  | | **Number of events in cell gate** | **HSC**  **CLEC12A+** | **MPP**  **CLEC12A+** | **MLP CLEC12A+** | **CMP**  **CLEC12A+** | **GMP**  **CLEC12A+** | **MEP**  **CLEC12A+** |
| **DONOR 1** | Events | 132407 | 0 | 0 | 1 | 502 | 331 | 29 |
|  | Parent events |  | 116 | 8 | 3 | 1201 | 414 | 253 |
| **DONOR 2** | Events | 669987 | 0 | 0 | 2 | 580 | 249 | 16 |
|  | Parent events |  | 173 | 28 | 10 | 1567 | 316 | 202 |
| **DONOR 3** | Events | 871671 | 1 | 0 | 0 | 642 | 289 | 18 |
|  | Parent events |  | 187 | 64 | 4 | 1763 | 365 | 326 |
| **DONOR 4** | Events | 761553 | 2 | 0 | 1 | 497 | 274 | 38 |
|  | Parent events |  | 103 | 46 | 7 | 2038 | 396 | 302 |
| **DONOR 5** | Events | 551661 | 2 | 0 | 0 | 231 | 227 | 17 |
|  | Parent events |  | 36 | 2 | 3 | 446 | 240 | 94 |
| **DONOR 6** | Events | 751663 | 0 | 0 | 0 | 121 | 78 | 20 |
|  | Parent events |  | 116 | 13 | 18 | 279 | 118 | 168 |
| **DONOR 7** | Events | 847546 | 2 | 0 | 1 | 725 | 605 | 53 |
|  | Parent events |  | 449 | 53 | 9 | 2446 | 773 | 514 |
| **DONOR 8** | Events | 325836 | 1 | 2 | 1 | 850 | 503 | 112 |
|  | Parent events |  | 325 | 137 | 20 | 3476 | 675 | 884 |
| **DONOR 9** | Events | 397753 | 0 | 0 | 0 | 268 | 172 | 47 |
|  | Parent events |  | 67 | 3 | 1 | 721 | 206 | 459 |
|  | % of parent |  | 0.0 | 0.0 | 0.0 | 37.2 | 83.5 | 10.2 |
| **DONOR 10** | Events | 381545 | 0 | 0 | 0 | 164 | 214 | 10 |
|  | Parent events |  | 24 | 2 | 0 | 256 | 234 | 130 |
| **DONOR 11** | Events | 512915 | 0 | 0 | 0 | 1150 | 2395 | 255 |
|  | Parent events |  | 340 | 125 | 13 | 3178 | 2883 | 1946 |
| **DONOR 12** | Events | 403093 | 0 | 0 | 0 | 497 | 605 | 103 |
|  | Parent events |  | 413 | 44 | 7 | 1061 | 675 | 930 |
| **DONOR 13** | Events | 1280958 | 0 | 0 | 0 | 2756 | 3441 | 989 |
|  | Parent events |  | 405 | 135 | 3 | 7918 | 4095 | 4440 |
