# Supplementary material for: Mapping the CLEC12A expression on myeloid progenitors in normal bone marrow; implications for understanding CLEC12A‐related cancer stem cell biology
Source: J Cell Mol Med. 2018 Feb 7;22(4):2311–8. doi: 10.1111/jcmm.13519 (PMC5867061; doi:10.1111/jcmm.13519)
Supplement: Supplementary file 3 [file JCMM-22-2311-s003.docx]

| **Table S2. Purity of FACS sorted subsets** | | | | | | | | | | | | |
| --- | --- | --- | --- | --- | --- | --- | --- | --- | --- | --- | --- | --- |
|  | **DONOR 1** | | **DONOR 2** | | **DONOR 10** | | **DONOR 11** | | **DONOR 12** | | **DONOR 13** | |
|  | Events/  events in cell gate | % | Events/  events in cell gate | % | Events/  events in cell gate | % | Events/  events in cell gate | % | Events/  events in cell gate | % | Events/  events in cell gate | % |
| **CMP CLEC12A+** | 30/33 | 90.9% | 44/77 | 59.5% | 194/202 | 96.0% | 106/114 | 93.0% | 21/63 | 33.3% | 105/110 | 95.5% |
| **CMP CLEC12A-** | 22/25 | 88.0% | ND | ND | 80/90 | 88.9% | 106/114 | 93.0% | 103/116 | 88.8% | 122/142 | 85.9% |
| **GMP CLEC12A+** | 74/77 | 96.1% | 42/53 | 79.2% | 33/37 | 89.2% | 151/157 | 96.2% | 12/12 | 100.0% | 80/92 | 87.0% |
| **GMP CLEC12A-** | ND | ND | ND | ND | ND | ND | 28/30 | 93.3% | ND | ND | ND | ND |
| **MEP CLEC12A+** | ND | ND | ND | ND | ND | ND | ND | ND | ND | ND | ND | ND |
| **MEP CLEC12A-** | ND | ND | ND | ND | 108/115 | 93.9% | 120/128 | 93.8% | ND | ND | 99/112 | 88.4% |
| *ND: not done, due to low cell numbers | | | | | | | | | | | | |
